# Hyperprogressive Disease After Immunotherapy: A Case Report of Pulmonary Enteric Adenocarcinoma

**DOI:** 10.3389/fonc.2022.799549

**Published:** 2022-03-07

**Authors:** Chun-Hong Hu, Shenghao Shi, Wen Dong, Lizhi Xiao, Hongjing Zang, Fang Wu

**Affiliations:** ^1^ Department of Oncology, The Second Xiangya Hospital, Central South University, Changsha, China; ^2^ Department of Oncology, The Changde First People’s Hospital, Changde, China; ^3^ Department of Radiology, Second Xiangya Hospital, Central South University, Changsha, China; ^4^ Department of Pathology, Second Xiangya Hospital, Central South University, Changsha, China

**Keywords:** pulmonary enteric adenocarcinoma, chemotherapy, immunity therapy, hyperprogressive disease, case report

## Abstract

Primary pulmonary enteric adenocarcinoma (PEAC) is a rare invasive adenocarcinoma clinically similar to metastatic colorectal adenocarcinoma (MCRC). Although many studies have addressed the differential diagnosis of PEAC, few have described the treatment of PEAC, especially using immunotherapy. This report describes a 61-year-old man who presented initially with pain in the ribs. Pathological analysis of biopsy samples shows malignant tumors of the right pleura, and next-generation sequencing of 26 genes showed a *KRAS* gene mutation. Positron emission tomography-computed tomography (PET-CT) found no evidence of gastrointestinal malignancy. Due to multiple metastases, the patient could not undergo radical surgery. The patient was treated with a combination chemotherapy regimen of paclitaxel plus carboplatin, along with sindilizumab immunotherapy, but, after one cycle of treatment, the tumor showed a hyperprogressive state. The patient is still being monitored regularly. These findings indicate that chemotherapy combined with immunotherapy may be ineffective in the treatment of primary PEAC with positive driver genes.

## Introduction

Pulmonary enteric adenocarcinoma (PEAC) is a very rare type of lung adenocarcinoma, first reported in 1991 (1). The 2015 World Health Organization (WHO) classification has defined PEAC as a primary lung adenocarcinoma containing more than 50% of intestinal differentiation components, with the tumor cells being positive for one or more immunohistochemical markers of gastrointestinal tumors. The pathogenesis of PEAC has not been fully determined, and there is currently no specific treatment plan. At present, the main treatment methods for PEAC are surgery and systemic chemotherapy. Although many case reports and studies have described the detection of different gene mutations in patients with PEAC, few have been administered targeted therapy (2).

The nonsynonymous tumor mutation burden (TMB) has been reported higher in patients with primary PEAC than in patients with ordinary lung adenocarcinoma, suggesting that patients with primary PEAC may be more likely to benefit from checkpoint blocking immunotherapy. Very few reports to date have described the effects of treatment with immunotherapy in patients with primary PEAC. The present report describes a patient with a rare primary lung and bowel adenocarcinoma who experienced tumor super-progression after chemotherapy combined with immunotherapy for primary PEAC.

## Case Description

**Figure 1:**
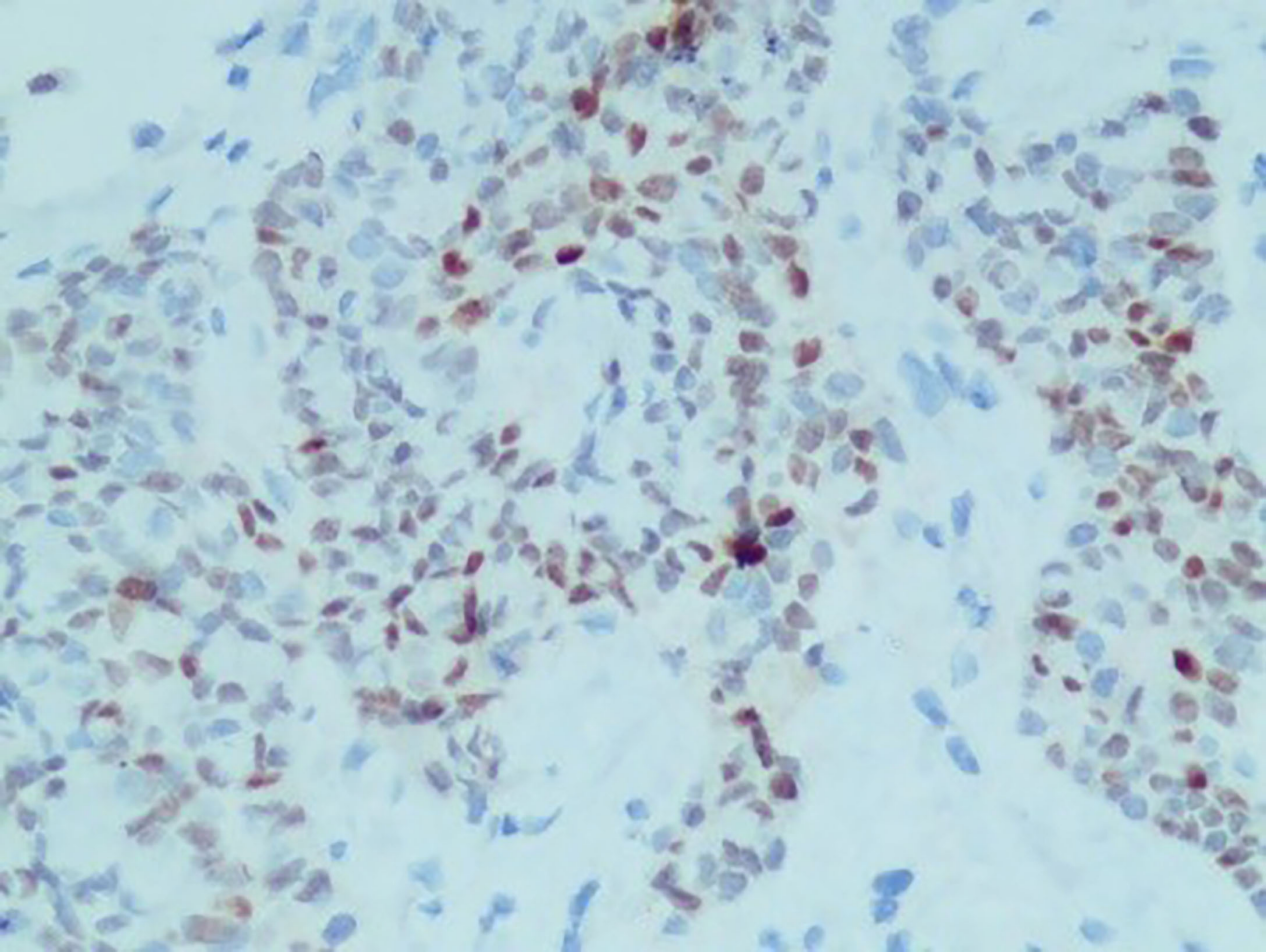
IHC: CDX-2 (+).

**Figure 2:**
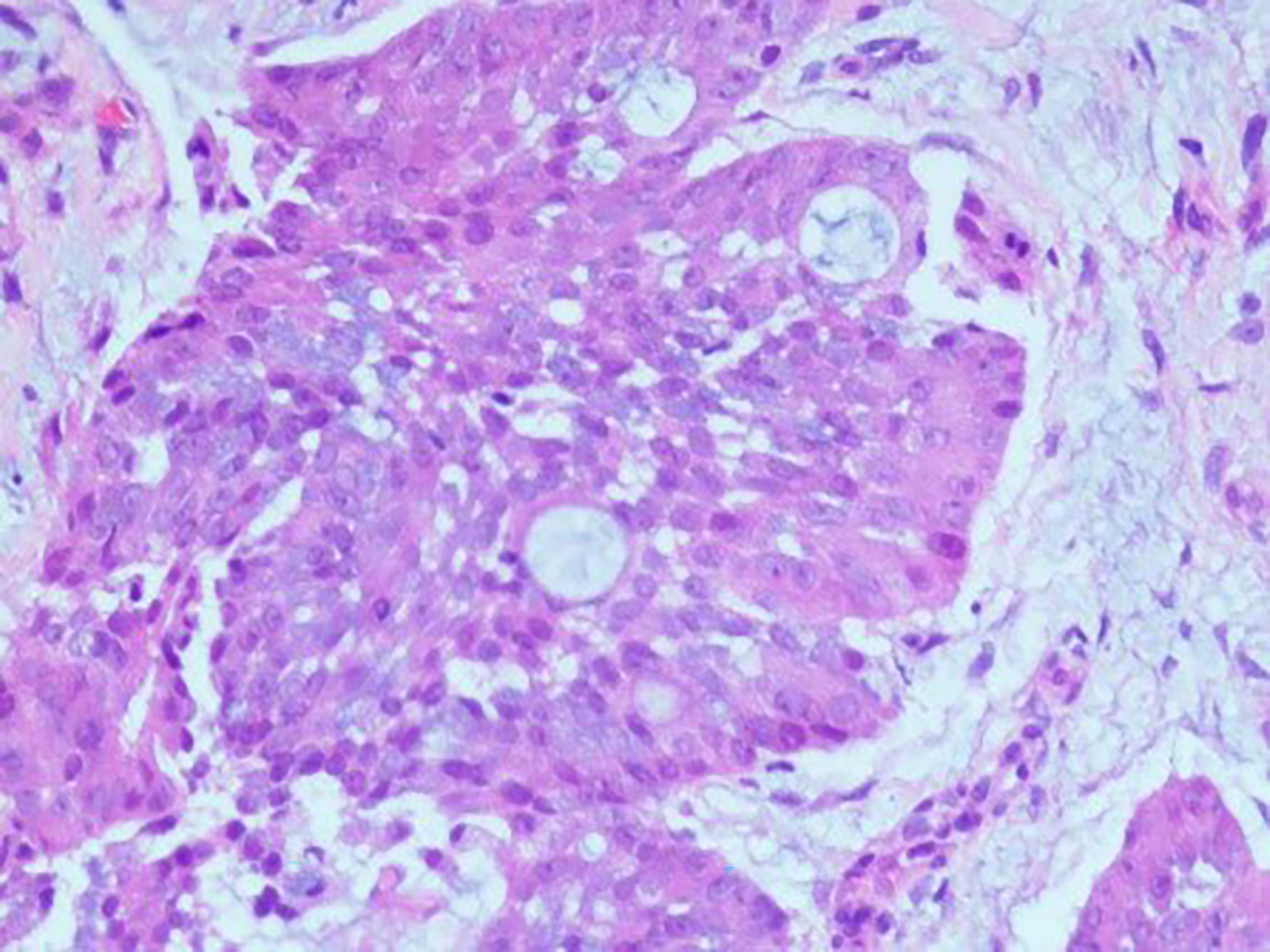
Pathology imagine.

A 61-year-old man visited the Second Xiangya Hospital of Central South University due to right chest pain for more than 4 months. In January 2021, he was seen at a local hospital; at that time, the patient reported having no obvious paroxysmal dull pain in the right chest or radiating pain between MS0-3. The patient had a dry cough, but no evidence of hemoptysis, hematemesis, chills, fever, or fatigue. Oral medication suggested by the local clinic was ineffective. At the beginning of March 2021, the patient visited a local hospital due to worsening pain on his right side, but there was no evidence of chills, fever, nausea, vomiting, dizziness, headache, abdominal pain, or diarrhea. The patient was admitted to the Department of Respiratory Medicine at a higher-level hospital on March 11, 2021, for further diagnosis and treatment. Physical examination at admission showed no obvious positive signs, and his ZPS score was 2 points. He was generally in good health and had no history of smoking or drinking. His parents died of unknown causes, although they had conditions similar to that of the patient. His family history included chronic diseases. His older sister died of “lung cancer,” and his younger brother had been diagnosed with “tongue cancer.” He reported no family history of genetic or infectious diseases. Computed tomography (CT) imaging of his lungs showed two lesions consistent with pneumonia and pleural effusion on his right side. Bronchoscopy showed multiple areas of mucosal irregularities in his visceral and parietal pleura, as well as multiple adhesive tapes between the visceral and parietal membranes. Examination of drained intracavitary fluid showed significant increases in tumor markers of pleural effusion, suggesting that this patient had malignant pleural effusion. Laparoscopy showed that the pleura occupied space, with the pathological diagnosis (**Figure 1**) being a malignant tumor in the right pleura. Immunohistochemical assays showed that the tumor was positive for CK7, CK19, and pd-l1 (CPS 5); focally positive for CDX-2 (**Figure 2**) and villin; and negative for napsin A and CK20. These findings, along with a thoracoscopic biopsy of his right chest showing a malignant tumor, were consistent with a diagnosis of mucoepidermoid carcinoma. Further pathological examination resulted in a diagnosis of a poorly differentiated intestinal-type adenocarcinoma of the right pleura. Next-generation sequencing (NGS) of 26 genes associated with lung cancer showed that this tumor was positive for a *KRAS* mutation (**Supplementary Materials**). Due to the possibility of metastasis, the patient underwent positron emission tomography (PET)-CT examination in our hospital. The results showed that the right pleura were extensively thickened and glucose metabolism had increased, findings consistent with membranous malignant tumors suggestive of metastases. In addition, the lymph nodes in the right hilum and mediastinum (zone 4) were slightly enlarged, with increased glucose metabolism, suggesting lymph node metastases. Sternal bodies and multiple thoracic and lumbar spine attachments were observed, with bones and right bone wings showing increased glucose metabolism and bone destruction, suggesting bone metastases. Gastrointestinal lesions, however, were not observed. These pathological and immunohistochemical findings excluded lung metastases of intestinal adenocarcinoma, and the patient was finally diagnosed with pulmonary intestinal adenocarcinoma.

**Figure 3:**
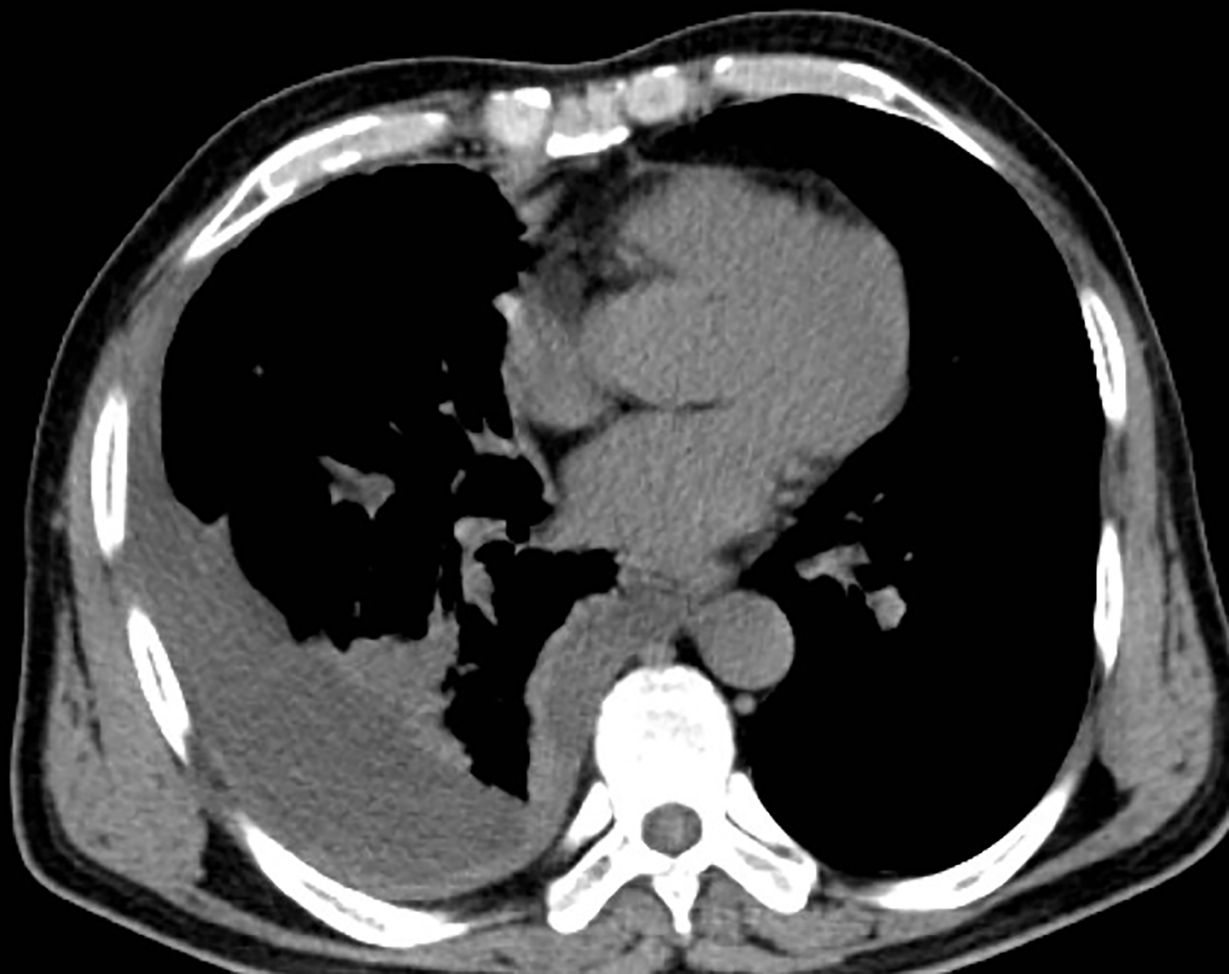
CT before immunotherapy.

**Figure 4:**
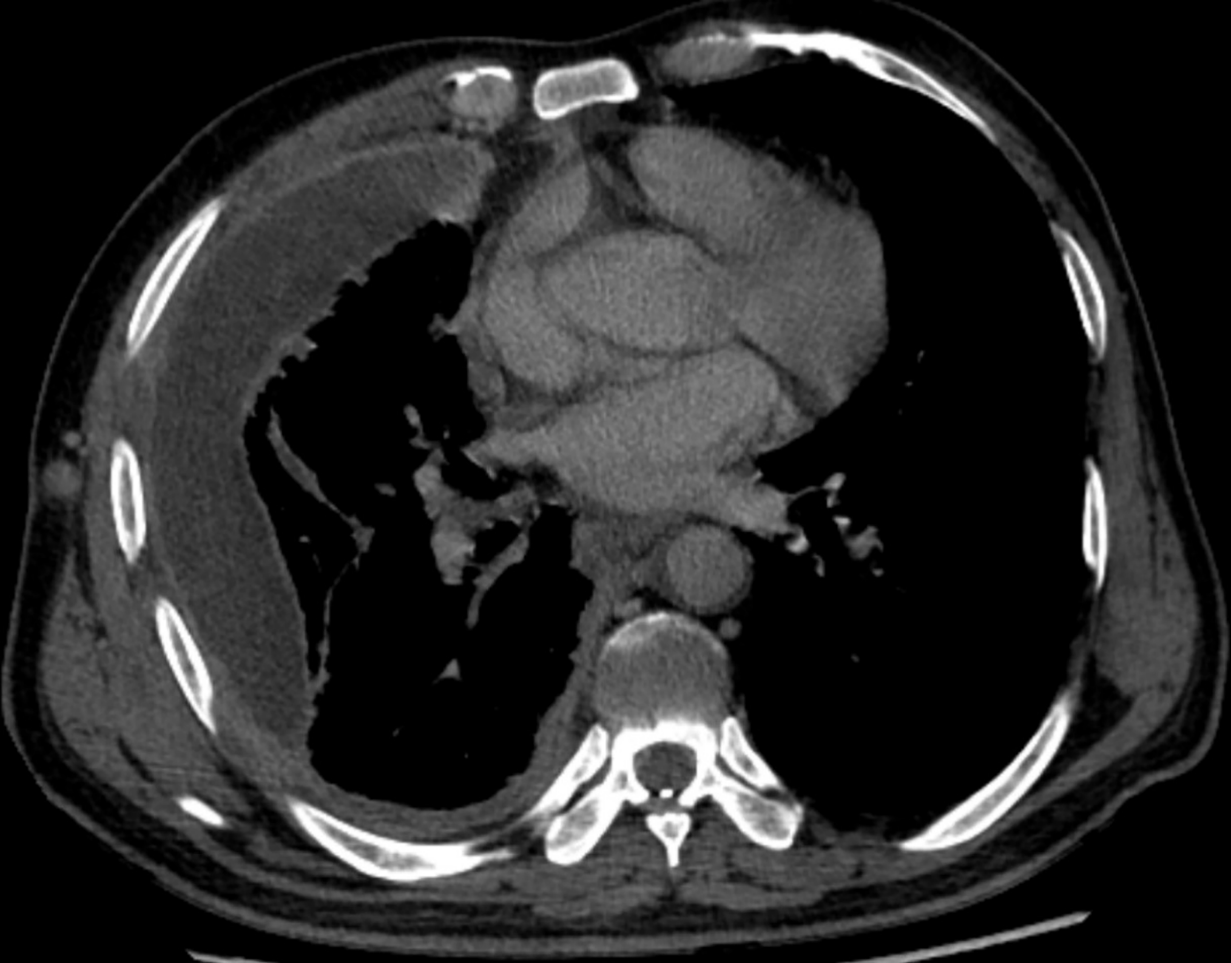
CT after immunotherapy.

As the patient had multiple metastases at presentation, radical surgery was no longer possible. Before the diagnosis, the patient received symptomatic treatment to relieve pain. After the diagnosis, the patient and his family were informed of the necessity of for antitumor therapy and its possible side effects, including myelosuppression, immune-related pneumonia, immune-related myocarditis, and liver damage. The patient and family members expressed their understanding and agreed to systemic chemotherapy plus sindilizumab immunotherapy. After one cycle of treatment, the patient reported poor pain control and a weight loss of about 3 kg. Repeat chest and abdominal CT showed significant thickening of the right pleura (**Figures 3**, **4**), indicating that the disease had progressed rapidly and that patient prognosis was extremely poor. Although a second-line treatment regimen was selected, consisting of paclitaxel combined with anlotinib, the patient and family members refused further treatment, as the patient was less likely to benefit from this regimen than from the first-line regimen. At this writing, the patient remains alive but in generally poor condition and continues to receive nutritional support at a local hospital.

## Discussion

In 2011, the International Association for the Study of Lung Cancer, the American Thoracic Society, and the European Respiratory Society jointly issued guidelines for the multidisciplinary classification of lung adenocarcinomas, enabling better treatment planning treatment and guiding prognosis (3). The treatment strategy for lung-intestinal adenocarcinoma, a rare type of lung adenocarcinoma, is similar to that for nonsmall cell lung cancer and can include various combinations of surgery, chemotherapy, radiotherapy, and targeted therapy (4). Patients with late stage tumors are ineligible for surgery can be treated successfully with four cycles of pemetrexed and carboplatin chemotherapy (5) or paclitaxel combined with cisplatin (6). Most lung and bowel adenocarcinomas are negative for EGFR mutations but positive for KRAS mutations (7), suggesting that tyrosine kinase inhibitors would be ineffective in these patients.

The 2021 Chinese Society of Clinical Oncology (CSCO) guidelines for the diagnosis and treatment of primary lung cancer have recommended immunosuppressants such as pembrolizumab as first-line treatment for patients with stage IV nonsquamous cell nonsmall cell lung cancer (NSCLC) without driver genes. PD-1 is highly expressed in PEACs, and nonsynonymous TMB is significantly higher in patients with primary PEAC than in patients with ordinary lung adenocarcinomas, suggesting that patients with PEAC may benefit from treatment with immune checkpoint inhibitors (8).

Although immunotherapy should have relatively good therapeutic potential in patients with lung-intestinal adenocarcinoma (9), the combination of immunotherapy and chemotherapy in the present patient resulted in tumor hyperprogression. Although hyperprogressive disease (HPD) after immunotherapy has not been defined precisely, it is thought to include (1) a ratio of tumor growth kinetic (TGK) variables during immunotherapy to pretreatment TGK >2 (10); (2) a tumor growth rate (TGR) ≥2-fold higher after than before immunotherapy (11); (3) the combination of a treatment failure time (TTF) <2 months, a >50% increase in tumor burden, and a TGR >2 after chemotherapy (12); (4) the combination of a TTF <2 months, a ≥50% increase in total diameter of the target lesions, and the appearance of at least two new lesions in the affected organs, a spread to new organs or worsened clinical manifestations, defined as a performance status (PS) ≥2 (13); or (5) progressive disease (PD) during the first 8 weeks of treatment, including a ≥40% increase in the total diameter of the target lesions, or the appearance of at least two new lesions in different organs (14). Based on all of these standards, the present patient developed HPD after only one cycle of chemotherapy combined with immunotherapy. A meta-analysis of 1,389 NSCLC patients included in six studies found that the incidence of HPD ranged from 8.02% to 30.43%, with overall survival rate being lower in patients with than without HPD. Risk factors for HPD included an Eastern Cooperative Oncology Group PS >1, a Royal Marsden Hospital score ≥2, a serum lactate dehydrogenase concentration above the upper limit of normal, more than two metastatic sites, and liver metastases (15). Other risk factors for HPD included age >65 years, gender, smoking history, neutrophil-to-lymphocyte ratio (NLR), PD1/PD-L1, PD-L1 status, monotherapy/combination therapy, and previous line of treatment. In contrast, HPD was not significantly associated with tumor number, histopathological type of NSCLC, EGFR mutation, KRAS mutation, or ALK rearrangement.

Hyperprogression in response to immunotherapy may have been due to driver gene mutations. For example, systemic chemotherapy treatment of a patient with advanced lung-intestinal adenocarcinoma and a new KRAS Q22K mutation resulted in rapid disease progression, with the patient dying within a short time (16). Although KRAS dependence may have been the main genetic factor responsible for poor patient prognosis, the small number of patients with this condition prevents definitive determination of the relevant mechanism.

In summary, a patient with a primary PEAC and a KRAS mutation experienced super-progression after being treated with immunotherapy combined with chemotherapy. Few reports to date have described the association between immunotherapy and outcomes in patients with lung-intestinal adenocarcinoma, especially in patients with treatment-associated HPD. The findings observed in this patient, including diagnosis, treatment, and the association between outcomes and driver genes may lead to studies on the treatment and prognosis of patients with lung and bowel adenocarcinoma.

## Data Availability Statement

The original contributions presented in the study are included in the article/**Supplementary Material**. Further inquiries can be directed to the corresponding author.

## Ethics Statement

Written informed consent was obtained from the individual(s) for the publication of any potentially identifiable images or data included in this article.

## Author Contributions

FW: were responsible for study conception and design and acquiring financial support. WD: provided patient information. SS: design, interpretation, and write-up of final article. All authors contributed to the article and approved the submitted version.

## Conflict of Interest

The authors declare that the research was conducted in the absence of any commercial or financial relationships that could be construed as a potential conflict of interest.

## Publisher’s Note

All claims expressed in this article are solely those of the authors and do not necessarily represent those of their affiliated organizations, or those of the publisher, the editors and the reviewers. Any product that may be evaluated in this article, or claim that may be made by its manufacturer, is not guaranteed or endorsed by the publisher.
