# Supplementary figures and images for: Hyperprogressive Disease After Immunotherapy: A Case Report of Pulmonary Enteric Adenocarcinoma
Source: Front Oncol. 2022 Mar 7;12:799549. doi: 10.3389/fonc.2022.799549 (PMC8937032; doi:10.3389/fonc.2022.799549)

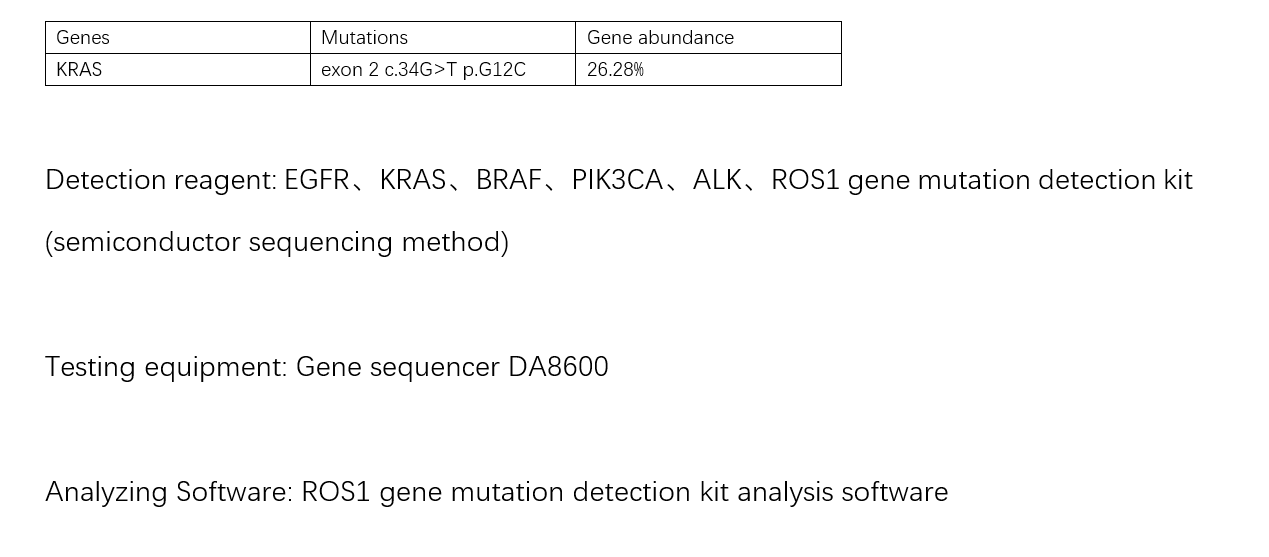

Supplement: Supplementary file 1 [file Image_1.png]
